# Effects of Filtering the Center of Pressure Feedback Provided in Visually Guided Mediolateral Weight Shifting

**DOI:** 10.1371/journal.pone.0151393

**Published:** 2016-03-18

**Authors:** Michael W. Kennedy, Charles R. Crowell, Michael Villano, James P. Schmiedeler

**Affiliations:** 1 Department of Aerospace and Mechanical Engineering, University of Notre Dame, Notre Dame, Indiana, United States of America; 2 Department of Psychology, University of Notre Dame, Notre Dame, Indiana, United States of America; Ludwig-Maximilian University, GERMANY

## Abstract

Thirty healthy adults completed a mediolateral weight-shifting balance task in which they were instructed to shift their weight to visually displayed target regions. A model-based filter and three different moving average filters employing 10, 34, and 58 samples were applied to the center of pressure visual feedback that guided the activity. The effects of filter selection on both the displayed feedback and the shift performance were examined in terms of shift time and non-minimum phase behavior. Shift time relates to feedback delay and shift speed, whereas non-minimum phase behavior relates to the force applied in shift initiation. Results indicated that increasing the number of samples in moving average filters (indicative of stronger filtering) significantly increases shift speed and shift initiation force. These effects indicate that careful selection and documentation of data filtering is warranted in future work and suggest opportunities for strategic filtering of visual feedback in clinical weight-shifting balance activities in order to improve outcomes based on such feedback.

## Introduction

Biofeedback in standing balance has been shown to result in improved efficacy and learning in balance therapy [1], though the relationship between biofeedback and balance performance is not fully understood [2–4]. The use of center of pressure (CoP) information to provide visual feedback (VFB) has been shown to improve balance control in healthy adults [5], and previous work has examined the effects of VFB design [6] on balance performance in healthy subjects. For individuals post-stroke, mediolateral balance specifically has been shown to be particularly indicative of balance performance [7]. In the rehabilitation of stroke, static balance tasks like quiet standing can be performed with or without VFB, while the dynamic balance task of weight shifting, commonly used in both clinical rehabilitation [8] and balance assessment [9], typically involves a subject being guided through a series of weight shifts with VFB in order to place his/her CoP within a displayed target region [7,10–13]. One motivation for performing mediolateral weight shifting tasks is that they allow information (such as time-to-target and path-planning data) to be collected from post-stroke dynamic balance performance, which is beyond the scope of static balance tasks [13].

**Fig 1:**
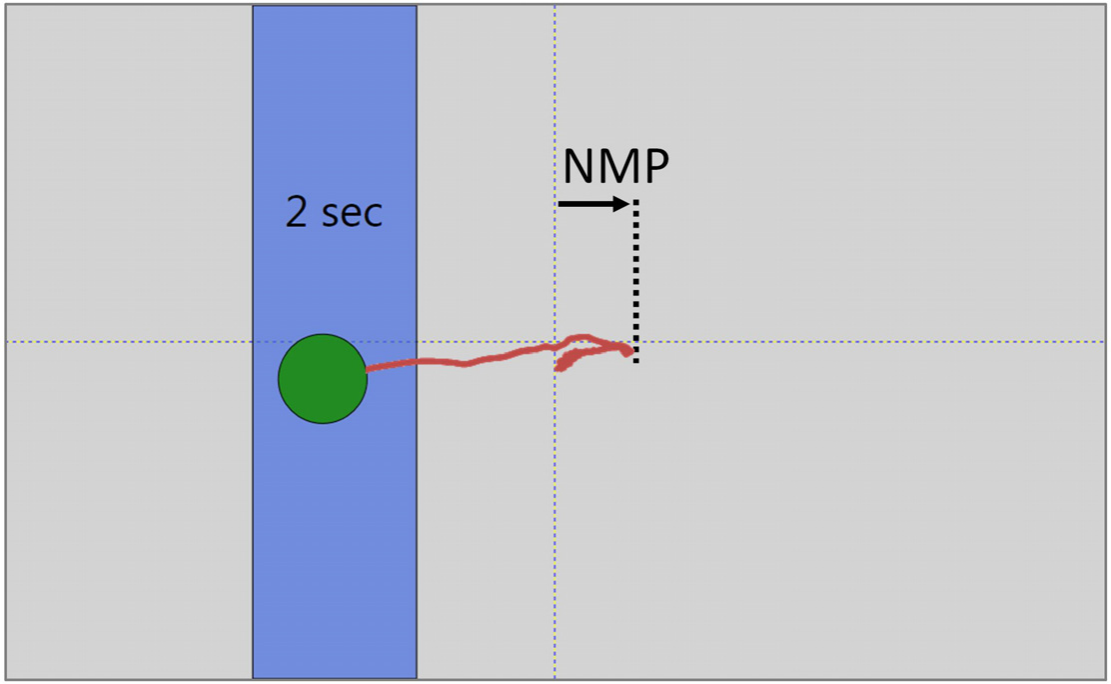
Non-Minimum Phase Behavior. As the subject shifts his/her position from the center to the blue target region on the left, his/her CoP first moves in the opposite direction due to the force required to initiate the weight shift, referenced in this and a previous study as NMP behavior. The red trace is not shown as a part of this study’s VFB.

Two variables change whenever a person’s weight is shifted. One is center of gravity (CoG), which is defined as the vertical projection of the person’s center of mass onto the ground [14]. The second is CoP, which is the location of the resultant force of the vertical support forces present in the person’s two legs [14]. Explicit measurement of CoG information requires motion capture equipment, whereas CoP information can be captured using a single force plate. The movements of the CoG and CoP during weight shifting are highly correlated except for a relatively brief period at the start of the shift during which the CoP exhibits a non-minimum phase (NMP) behavior signified by its movement in a direction opposite that of the weight shift [15] (Fig 1). The mechanical forces that are generated by torques applied through the hips and ankles and are needed to initiate a shift of weight result in this NMP behavior, thus causing the CoP to follow a biphasic path, first opposite and then aligned with the direction of the weight shift. In contrast, the CoG position typically changes monotonically in the same direction throughout the shift.

Because a single force plate is a convenient way to instrument balance measurement, most studies involving dynamic balance activities have recorded and examined the CoP rather than the CoG during weight shifting. Likewise, when VFB is provided in these studies, it is based on the CoP, not the CoG. Under these circumstances, the presence of the NMP behavior could potentially be confusing if people observe that their displayed CoP initially moves opposite to the intended shift direction. In normal practice, such confusion is mitigated at least somewhat by filtering the displayed VFB. This filtering is generally performed to reduce feedback noise and is achieved through the use of a smoothing function applied to the raw CoP measures provided by the force plate. The presence of smoothing is likely to obscure some of the NMP behavior in the VFB, thereby making the displayed CoP path appear to be more nearly monotonic.

Few studies have fully reported specifications for filters applied to the provided VFB [6,7,16]. While one type of filtering (addition of time delay to VFB) has been shown to alter static balance performance [17] through smoothing that decreases the difference between the CoP and the estimated CoG position, the effects of filtering on dynamic balance performance have not been previously explored. In this study, moving average filters were applied to CoP VFB in the context of mediolateral weight shifting. Use of this simple type of filter provides a basis for adjusting VFB in a straightforward manner, while potentially affecting balance performance. Applying a simple moving average filter to the CoP data smooths it, reducing the magnitude of the visually displayed NMP behavior and thereby making the visually displayed CoP position more similar to that of the actual CoG position early in a weight shift. Such filtering, however, naturally results in VFB delay, which can cause greater differences between the CoP VFB and the actual CoG positions later in a shift. Candidate approaches to reduce this difference without the addition of VFB delay are postulated in the Discussion.

This study examines VFB provided with significantly different magnitudes of moving average filtering, as well as a model-based filter, in order to examine the effects on both the provided VFB and the resulting subject balance performance. It is hypothesized that increasing filter magnitude results in increased time required for the VFB to shift to each target and reduced magnitude of the NMP behavior displayed in VFB, resulting in significant differences in physical shift performance. The objective is to develop a more fundamental understanding of VFB implementation in dynamic balance tasks, as well as to inform the design of future VFB systems.

## Methods

### Ethics Statement

All subjects gave their informed consent prior to inclusion in the study, and this research received approval from the Notre Dame Institutional Review Board (Protocol ID: 12-06-375).

### Subjects

This study incorporated data from 30 healthy subjects, who consisted of 7 males and 23 females, ranging from 18 to 21 years of age (body mass 63.9 ± 11.3 kg; height 170.7 ± 8.0 cm; this form indicates mean ± standard deviation, as it does for all data presented subsequently). All reported having no visual or balance impairments that could have led to difficulty in performing the weight-shifting tasks in this study; however, independent confirmation of their balance capabilities was not obtained.

### Experimental System

The WeHab system [18] was employed by a researcher to lead all subjects through the research protocol. This system consists of a computer running the WeHab software, two Nintendo Wii Balance Boards for data collection, one Nintendo Wii remote for researcher input, and two webcams for collecting visual data. Balance Board data were collected at a sampling rate of 63.4 ± 1.4 Hz. Apart from the use of a small desktop computer instead of a laptop computer, the WeHab setup used in this study was identical to that from previous work [6]. While being led through each balance task, each subject stood with one foot on each of the two Nintendo Wii Balance Boards placed with 31.8 cm separation between their centers. A chair located directly behind the Balance Boards provided the place for the subject to rest between activities without the subject needing to move his/her feet. During the study, a researcher sat in a nearby chair, read instructions to the subject, and used a Nintendo Wii remote to advance through each activity. A 27-inch monitor providing VFB was located a horizontal distance of 134 cm away from the front of the Balance Boards, and its height was adjusted to the eye level of each subject by the researcher.

### Visual Feedback

**Fig 2:**
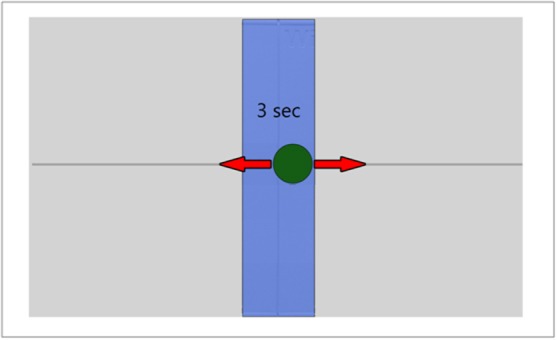
Visual Feedback. Feedback provided to each subject during the study. As the subject’s position shifted left or right, the green CoP marker shifted left or right. The green CoP marker’s vertical position was fixed. The blue rectangle represents the target region that the subject is attempting to reach with his/her CoP, while the time display indicates how long the CoP has remained within the target region. The arrows added to the figure indicate the directions of marker movement and were not part of the visual feedback.

For all VFB conditions, the display components of the VFB were the same. One-dimensional direct CoP VFB was provided by a green circular marker placed on a grey planar background denoting the subject’s (filtered) mediolateral CoP location (Fig 2). The horizontal position of the marker denoted this CoP location, while the vertical position of the marker was fixed to the horizontal axis at the middle of the display.

CoP data were calculated from vertical reaction force data obtained from the Balance Boards. Each Balance Board consists of force transducers that provide vertical force information for each of the four quadrants of the device [19]. The mediolateral CoP location for a subject standing on two Balance Boards can be computed as

$$x_{CoP}=\frac{F_{R}-F_{L}}{F_{R}+F_{L}}d_{x},$$

where *F*_*R*_ and *F*_*L*_ are the total forces present on the right and left Balance Boards, respectively, and *d*_*x*_ is a multiplier that converts the vertical force ratio into a mediolateral distance. In this study, a value of 0.159 m (half the distance between the two feet) was used for *d*_*x*_. From this equation, a subject’s CoP can range from −*d*_*x*_ to *d*_*x*_, where a value of 0 represents equal weight distribution between both legs.

**Fig 3:**
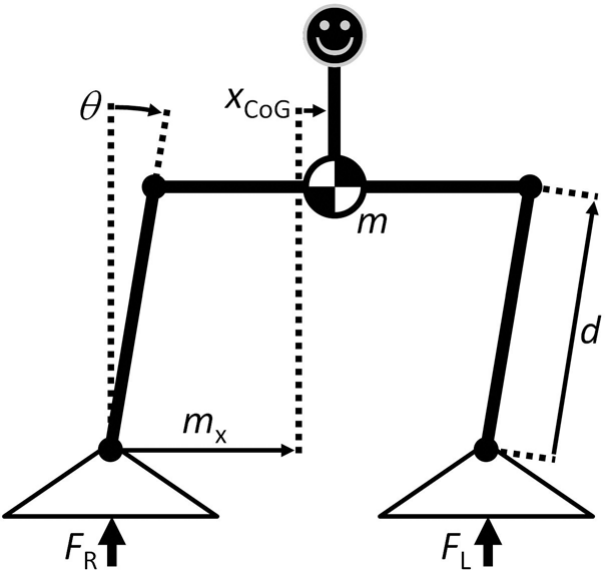
Parallelogram model. Model used for calculations for the CoP_Mod_ feedback condition. In this model, *θ* is the estimated angular deviation of the subject’s CoM from neutral stance, *m* is the subject mass, *x*_*CoG*_ is the lateral position of the CoM, *F*_*R*_ is the vertical force exerted on the right foot, *F*_*L*_ is the vertical force exerted on the left foot, *d*_*x*_ is half the lateral distance between the centers of the two feet, and *d*_*y*_ is the approximate distance between the subject’s hip and ankle (0.491 × subject height [23]).

In quiet standing, the CoG and CoP trajectories share an average value [14] and are in-phase [20]. Previous studies have used CoP data from quiet standing to estimate the CoG through use of a low-pass filter [21,22]. In a dynamic weight-shifting balance task, the difference between the CoG and the CoP is more readily apparent, and this low-pass filter method does not produce accurate CoG trajectories [22]. This inaccuracy motivated the use of a model-based VFB condition (CoP_Mod_) in the present study in the form of an estimate of the mediolateral CoG position computed by numerically integrating the dynamic equation of motion for a parallelogram model (Fig 3),

$$\ddot{\theta }=\frac{d_{x}(F_{R}-F_{L})-mgd_{y}\theta -2d_{y}m\sqrt{gd_{y}}\dot{\theta }}{md_{y}^{2}},$$

where *θ* is the estimated angular deviation of the subject’s center of mass from neutral stance, *m* is the subject mass, *g* is the acceleration due to gravity, and *d*_*y*_ is the approximate distance between the subject’s hip and ankle (0.491 × subject height [23]). This model assumes that all of the subject’s mass is concentrated at a central point (approximated at hip level) and that the subject’s upper body remains vertical and without arm movement. The equation of motion is linearized about neutral stance (*θ* = 0) using the small angle approximation, and the human postural control system is assumed to achieve a critically damped response [24] (this defines the $\dot{\theta }$ coefficient in the linearized equation of motion).

Along with the CoP_mod_ condition, three additional VFB conditions using moving average filters were examined. The first (CoP_10_) was calculated using a ten-sample (0.14 ± 0.01 s) moving average filter to reduce noise effects in the raw CoP data, as consistent with previous work [6,18]. The second (CoP_34_) and third (CoP_58_) moving average VFB conditions were similar to the first, except they incorporated thirty-four and fifty-eight samples (0.53 ± 0.02 s and 0.91 ± 0.02 s, respectively) in their moving average filters. The number of samples in the CoP_58_ filter was selected to result in VFB delay comparable to the modeled VFB condition (CoP_Mod_), while the CoP_34_ filter was designed to split the difference between the CoP_10_ and CoP_58_ conditions.

### Procedure

As in [6], subjects stood with their feet parallel on two Balance Boards, with one board centered under each foot. Each board was positioned with the long axis aligned with the length of the subject’s foot and the Balance Board LED indicator facing outward. Subjects were instructed to keep their arms at their sides throughout the study to reduce body motion due to upper-limb activity. All subjects wore comfortable shoes in order to provide consistency.

Subjects experienced the four VFB conditions in one of four sequences (CoP_10_, CoP_Mod_, CoP_58_, and CoP_34_; CoP_34_, CoP_10_, CoP_Mod_, and CoP_58_; CoP_58_, CoP_34_, CoP_10_, and CoP_Mod_; and CoP_Mod_, CoP_58_, CoP_34_, and CoP_10_) generated using a Latin square. A sequence was randomly assigned to each subject, with some constraints applied to ensure appropriate distribution of the sequences across the subjects. At the beginning of testing with each of the four VFB conditions a subject experienced in his/her assigned sequence, visual instructions were provided on the monitor as follows: “Quickly shift your weight to each blue target when it appears. Targets will alternate between center and a randomized side. Time will start once you are centered for 3 s." In all cases, a trial began only after the subject had maintained his/her weight in a neutral stance for 3 s (as assisted by VFB). For each VFB condition, the first trial was a short demonstration consisting of eight shifts (four shifts to offset target positions, each followed by a shift back to a central target corresponding to neutral stance) accompanied by the appropriate VFB. This demonstration trial served to ensure that the subject was familiar with the current VFB condition, as consistent with prior studies [5–7], before performing the actual weight-shifting balance task in trials two through four.

The second, third, and fourth trials within each VFB condition consisted of 20 shifts each. As in [6], the displayed target regions alternated between a central target (requiring equal weight across both feet) and an offset target (centered at a location requiring a 70%-30% distribution of the subject’s weight across both feet). The offset target location was randomized between the left and right sides, and each of the ten shifts to offset target positions was followed by a shift back to the central target. Unlike in [6], each trial ended not when a timer expired, but when 20 shifts were completed; this was done to encourage faster shifting and to ensure that an equal number of shifts were captured for each trial. Additionally, the hold time (time required for the subject to remain within a target region before the next target region appeared) was randomized between 2 and 4 s for each target. The target hold timer was reset if the subject left the target region before the hold time was over. Both the random hold time and the randomization of direction were intended to prevent anticipation effects, where subjects would begin to shift in the direction of the next predicted target location before the target region had actually changed position.

After each trial, including the demonstration trial, the subject was instructed to sit down for 45 s of rest to mitigate any potential physical fatigue effects. During this rest time, subjects sat in a provided chair and rested until the break was complete. In order to gauge any physical or mental fatigue effects that were present despite these breaks, subjects were asked afterwards whether they felt tired during the study. With each subject completing four trials (68 lateral weight shifts) for each of four VFB conditions, the total duration of each session, including rest, was about 38 minutes on average.

### Metrics

This study examined weight shifting performance in terms of the metrics time-to-target and magnitude of NMP behavior [15]. With the introduction of filtered VFB, the motion of the CoP marker visually presented to the subject generally differs from his/her actual measured CoP motion. Therefore, there are two relevant values for each metric–one quantifying the physical shift performance by means of the actual CoP motion and the other quantifying the performance visually fed back to the subject by means of the filtered CoP marker’s motion. All four metrics were log-transformed to improve normality.

The time-to-target metric for the physical shift, *t*_*CoP*_, is the time required for the subject’s CoP, computed with a 10-sample moving average filter applied, to first reach the specified target region. (This filter has been used as the baseline CoP VFB in previous studies [6,18] and was tuned to incorporate just enough smoothing to eliminate noise from the raw data.) Smaller *t*_*CoP*_ values indicate quicker shift speeds and superior weight-shifting performance [7]. Time *t* = 0 for computing *t*_*CoP*_ is referred to as the reaction instant, meaning the time at which the subject recognized the new target location and began shifting his/her weight [15]. As in [15], this instant was identified through use of a three-sample moving window that iterated backward from the time of maximum displacement of the CoP in the direction opposite the target (the time of maximum NMP behavior); when the window no longer contained a point closer to the target location than any point previously examined, the point with the last minimum distance was taken to mark the reaction instant. The time-to-target metric for the VFB presented to the subject, *t*_*VFB*_, is the time required for the subject’s VFB indicator to first reach the specified target region. The difference between *t*_*VFB*_ and *t*_*CoP*_ indicates the relative delay introduced by the VFB condition. Note that the CoP_10_ condition introduces no delay by this definition since the 10-sample moving average filter is applied for the calculation of *t*_*CoP*_. The reaction instant marking the time *t* = 0 for computing *t*_*VFB*_ was identified in the same manner as it was for computing *t*_*CoP*_.

The magnitude of the NMP behavior for the physical shift, *r*_*NMP*,*CoP*_, is the distance the subject’s CoP, again computed with a 10-sample moving average filter applied, travels in the direction opposite the weight shift. Larger values of *r*_*NMP*,*CoP*_ indicate more forceful shift initiation [15]. The CoP’s position at the reaction instant, referred to herein as the initial CoP position, serves as the reference position from which *r*_*NMP*,*CoP*_ is computed as $\frac{d_{NMP,CoP}}{d_{shift,CoP}}$, where *d*_*NMP*,*CoP*_ is the mediolateral distance from the initial CoP position to the CoP trajectory’s farthest point from the target and *d*_*shift*,*CoP*_ is the distance from the initial CoP position to the center of the target region. The magnitude of the NMP behavior observable by the subject via VFB, *r*_*NMP*,*VFB*_, is calculated in a similar manner, $\frac{d_{NMP,VFB}}{d_{shift,VFB}}$, where *d*_*NMP*,*VFB*_ is the mediolateral distance from the initial VFB indicator’s position (identified at the reaction instant computed from the filtered CoP trajectory) to the point on the VFB indicator’s trajectory farthest from the target and *d*_*shift*,*VFB*_ is the distance from the initial VFB indicator’s position to the center of the target region. The difference between the *r*_*NMP*,*VFB*_ and *r*_*NMP*,*CoP*_ metrics indicates the attenuation of the NMP behavior shown to the subject through VFB accompanying a weight shift.

### Statistical Analysis

Statistical analysis was performed through linear mixed models using SAS version 9.4 (SAS Inc., Cary, NC). Subject and VFB sequence were specified as random factors. Initial analysis of the effects of target location (center vs. offset) were analyzed; based on these results, the final analysis was performed separately on both center and offset target data subsets. The following main effects were examined:

- Feedback–Form of provided VFB (CoP_10_, CoP_34_, CoP_58_, or CoP_Mod_);
- Order–Ordinal position of the given VFB conditions (range: 1–4).

Gender has been shown not to have a significant effect on dynamic balance performance [6] and was not examined as a main effect in this study.

As in [6], main effects were examined using a stepwise selection process incorporating both forward selection and backward elimination [25] using an upper limit of p = 0.15. The final linear mixed models were then evaluated for each data set. When significant order effects were found, additional analysis was performed on subsets of the data. The type I error threshold was set at p = 0.05. Dependent variables were checked for normality using the SAS software’s univariate procedure. Analysis was performed using individual weight shift data. Pearson correlation coefficients were calculated using the SAS software’s CORR procedure. Due to the incorporation of multiple main and random effects into the linear mixed models used for calculation of statistical p-values, the shown graphs of average metric values with standard deviations may not provide an intuitive demonstration of statistical difference between VFB conditions.

## Results

Data from 5 subjects who reported feeling tired and having difficulty focusing on the task were excluded from the analysis. Also, weight shifts with metric values outside of three standard deviations of the mean (5.5% of all shifts) were excluded. For both NMP metrics, shifts with values equal to 0 (0.04% of all shifts) were also excluded to remove shifts affected by anticipation. Mixed model analysis of the remaining dataset with both central and offset target locations showed that target location was a significant factor for all four balance metrics (*F* (1, 5074) = 729.65, *p*<0.0001). These results indicated that central targets were associated with faster shifts and higher NMP behavior, perhaps because subjects could at least anticipate the direction of the shift with central targets, if not the timing of the appearance of the central target. Note that an offset target was always followed by a central target (neutral stance), whereas from a central target, the next offset target could appear on either side. In order to avoid these directional anticipation effects, results from shifts only to offset target locations are examined and discussed below.

No statistically significant differences were found between the CoP_Mod_ and CoP_58_ VFB conditions across all four metrics. Therefore, results for the CoP_Mod_ VFB condition are not discussed further. Finally, significant order effects for both *t*_*CoP*_ (*F* (3, 2196) = 6.87, *p*<0.0005) and *r*_*NMP*,*CoP*_ (*F* (3, 2196) = 5.21, *p*<0.005) showed that the first feedback condition resulted in smaller *t*_*CoP*_ and larger *r*_*NMP*,*CoP*_ than the later feedback conditions. As such, results from later feedback conditions are the primary focus of this paper’s discussion. A total of 1,635 shifts were included in the data analysis of the CoP_10_, CoP_34_, and CoP_58_ VFB conditions for VFB in the 2^nd^, 3^rd^, and 4^th^ positions of the counterbalanced sequences. A summary of the resulting effects of VFB filtering on *t*_*VFB*_, *t*_*CoP*_, *r*_*NMP*,*VFB*_, and *r*_*NMP*,*CoP*_ are shown in Table 1.

**Table 1:** Table of Results.

	VFB	CoP
**Time**	**+**	**-**
**NMP**	**-**	**+**

The effects of increased VFB filtering on *t*_*VFB*_ (top left), *t*_*CoP*_ (top right), *r*_*NMP*,*VFB*_ (bottom left), and *r*_*NMP*,*CoP*_ (bottom right).

+: Statistically significant increase associated with increasing VFB filtering

-: Statistically significant decrease associated with increasing VFB filtering.

### VFB Indicator Results

**Fig 4:**
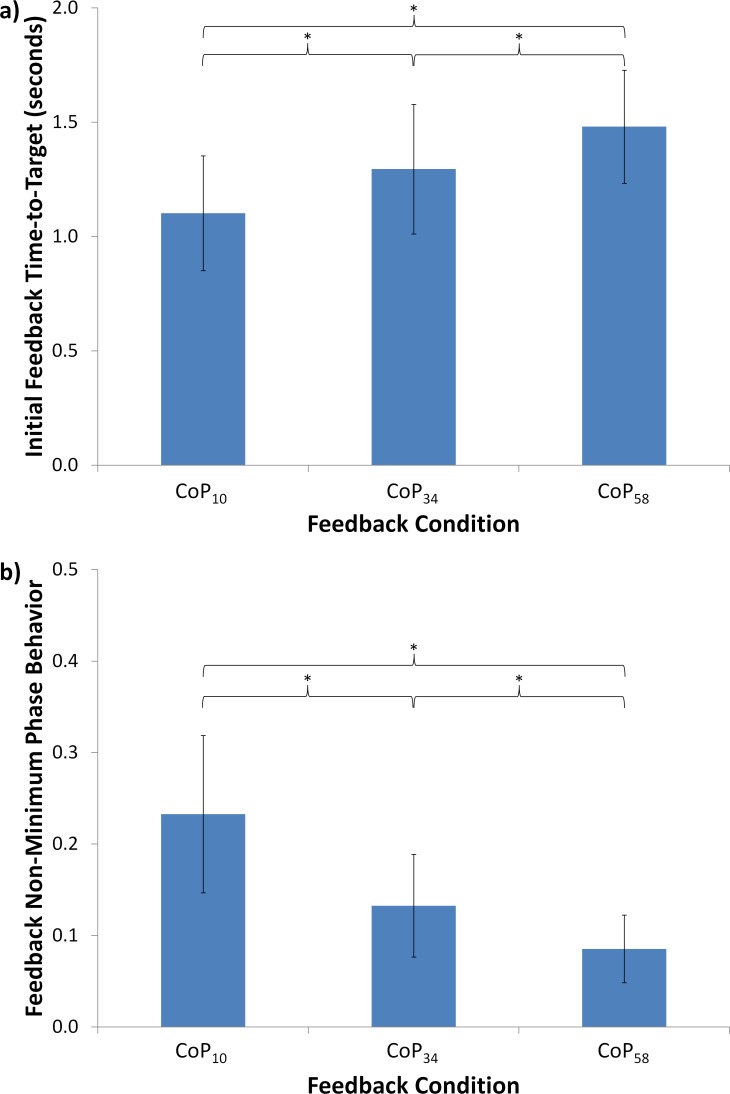
VFB Performance Results. Average values for VFB indicator performance based on (a) initial feedback time-to-target and (b) feedback non-minimum phase behavior across the three filtered feedback conditions. Bars indicate one standard deviation. *: p<0.0001.

The mixed models for both *t*_*VFB*_ and *r*_*NMP*,*VFB*_ as determined using stepwise selection consisted of feedback alone. Results for *t*_*VFB*_ due to feedback condition are shown in Fig 4A. The feedback condition effect for *t*_*VFB*_ was significant (*F* (2, 1603) = 379.5, *p*<0.0001), with increased VFB filtering being associated with increased *t*_*VFB*_ metric values. Equivalently, in addition to the smoothing effect, increased filtering resulted in significant increases in VFB delay, with a difference of 0.38 s (34% increase) between the CoP_58_ and CoP_10_ VFB conditions.

Results for *r*_*NMP*,*VFB*_ due to feedback condition are shown in Fig 4B. Again, the feedback effect was significant (*F* (2, 1603) = 759.06, *p*<0.0001), with increased VFB filtering being associated with decreased *r*_*NMP*,*VFB*_ metric values, or equivalently, increased filtering significantly decreased the NMP behavior that was visually displayed to subjects. The CoP_34_ VFB condition resulted in an average of 43% less NMP behavior shown to subjects on the display compared to the CoP_10_ VFB condition, and the CoP_58_ VFB condition resulted in an average of 63% less NMP behavior shown.

### Shift Performance Results

**Fig 5:**
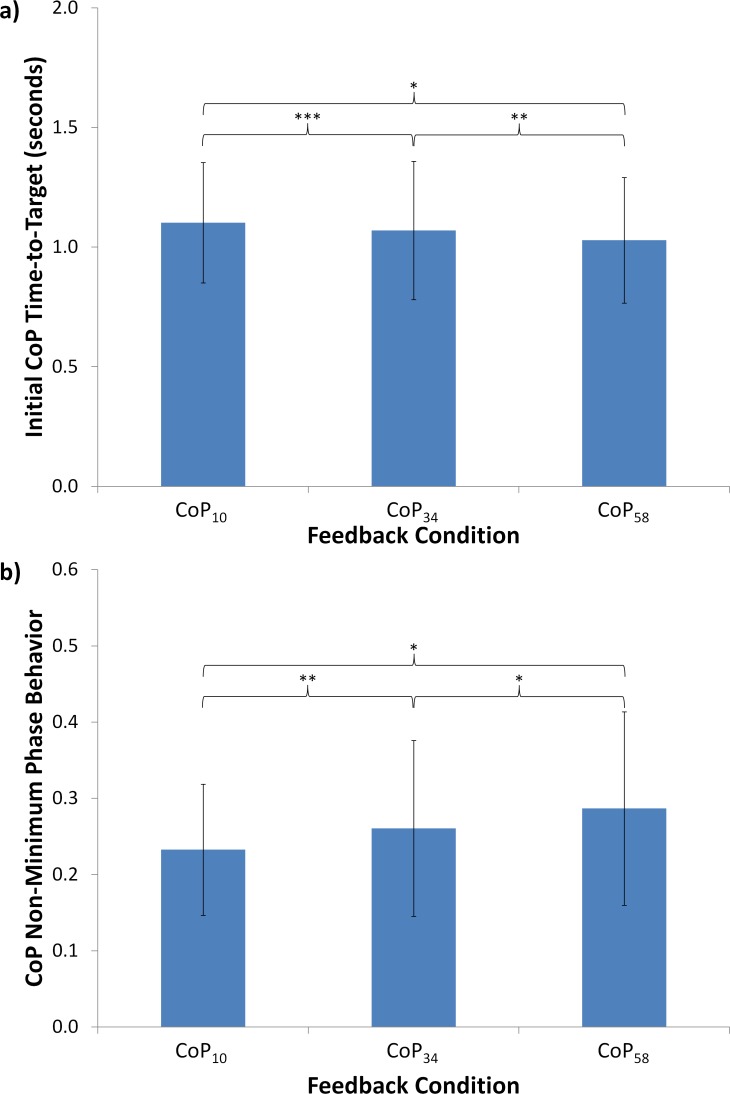
Shift performance results. Average values for shift performance based on (a) initial CoP time-to-target and (b) CoP non-minimum phase behavior across the three filtered feedback conditions. Bars indicate one standard deviation. *: p<0.0001; **: p<0.01; ***: p<0.05.

The model for *t*_*CoP*_ consisted of both feedback (*F* (2, 1601) = 13.11, *p*<0.0001) and ordinal feedback condition (order; *F* (2, 1601) = 2.88, *p*>0.05). Results for *t*_*CoP*_ due to feedback condition are shown in Fig 5A. Increased VFB filtering was associated with significantly decreased *t*_*CoP*_ metric values (0.07 s or 7% decrease between CoP_10_ and CoP_58_). The model for *r*_*NMP*,*VFB*_ consisted of feedback alone (*F* (2, 1603) = 33.88, *p*<0.0001). Results for *r*_*NMP*,*CoP*_ due to feedback condition are shown in Fig 5B. Increased VFB filtering was associated with increased *r*_*NMP*,*CoP*_ metric values. For all three VFB conditions, *t*_*CoP*_ and *r*_*NMP*,*CoP*_ demonstrated negative correlations of intermediate magnitude (-0.4322, -0.5958, and -0.6500 for CoP_10_, CoP_34_, and CoP_58_, respectively).

### NMP Behavior Results

**Fig 6:**
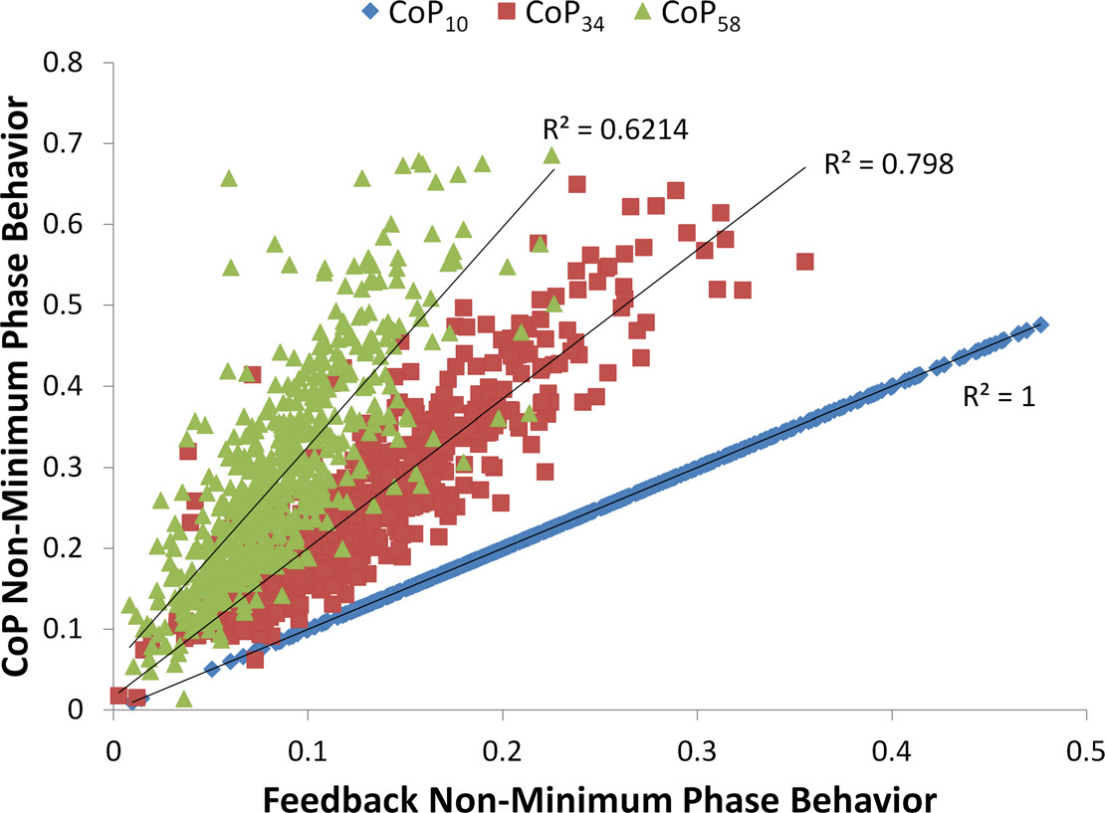
Feedback vs. CoP NMP results. Relationship between feedback NMP behavior and CoP NMP behavior for all three filtered feedback conditions. Trendlines and R^2^ values are shown for each feedback condition.

Fig 6 plots *r*_*NMP*,*CoP*_ versus *r*_*NMP*,*VFB*_ and includes linear fit trend lines for each VFB condition. By definition, the two metrics are identical in the CoP_10_ case, so the corresponding slope is simply unity (p-value = 0). The trend line slopes for the CoP_34_ and CoP_58_ cases are 1.8 (p-value < 0.005) and 2.7 (p-value < 0.001), respectively. These values indicate that the relationship between CoP NMP behavior and VFB NMP behavior is altered across the three VFB conditions.

## Discussion

### Filtering increases VFB delay and decreases visually displayed NMP behavior

The VFB indicator results demonstrate that the different filters and the smoothing they provided were sufficient to provide distinct VFB conditions in terms of both VFB delay and visually displayed NMP behavior, which increased and decreased, respectively, as the size of the moving average filter window increased. The filters’ attenuation of visually displayed NMP behavior early in a weight shift could be desirable since it reduces the discrepancy between the desired and actual initial movement of the visual indicator. The relatively large VFB delay that accompanies this attenuation is problematic, though. Throughout the latter course of the weight shift, the delay creates a discrepancy between the visual indicator’s position and the subject’s own sense of his/her progress toward completing the shift. Previous studies have shown that the addition of VFB delay in static balance, wherein it decreases the difference between CoP and estimated CoG position [17] (the same is true only early in a weight shift, with the opposite occurring later), leads to a decrease in subject reliance on the VFB in young healthy subjects [26] and increased sway in elderly healthy subjects [27]. Taken together, these results suggest that significant VFB delay is likely undesirable for weight shifting activities. Potentially reducing subject reliance on the VFB that is essentially required to define the task they are asked to perform would be counter-productive unless an alternative form of feedback, such as auditory, was being emphasized instead. In most cases then, the magnitude of VFB filtering for weight shifting tasks should be selected based on the effects of filtering on actual weight-shifting performance discussed below.

### Filtering increases shift speed and NMP behavior

The observed decreases in *t*_*CoP*_ associated with increased filtering are consistent with the subjects performing the weight-shifting task by controlling their CoG rather than explicitly trying to control the visually displayed CoP marker, at least through the initial movement of the CoP into the target region. Had subjects sought to control the CoP marker’s position explicitly, one would have anticipated physical shift times to be positively correlated with VFB delay due to subjects reducing their shift speeds (and thus increasing their shift times) with the increase in VFB delay. These results suggest that subjects may indeed have transferred reliance from the VFB associated with the balance task to balance information obtained through proprioception, vestibular input, and/or visual input from the surroundings as VFB delay increased (as predicted by [26]). Regardless, increasing filtering, at least up to a moving average of 58 samples, is an effective means to encourage young, healthy subjects to perform quicker weight shifts with stronger shift initiation forces. The effects of VFB delay could potentially be quite different for older subjects with or without pathological balance and thus, warrants further examination in future studies as discussed later in this section.

If reduced reliance on the VFB does explain the quicker shifts associated with increased filtering, that would suggest that a subject’s own internal assessment, based on other sensory inputs, of what constitutes “quickly shifting” differs somewhat from his/her interpretation of the VFB when he/she does rely on it. Evaluating this phenomenon would require careful experimental design since, unlike static balance, weight shifting requires some feedback to inform the subject that the desired offset CoP position has been achieved. One possibility would be an implementation of VFB in which the CoP marker is only visible when located within a target region; this would enable use of VFB to inform subjects as to their CoP location in a static sense, while allowing the subject to rely on proprioception, vestibular input, and other visual input along with the location of the visually displayed target region to accomplish the dynamic weight shift.

As shown by the *r*_*NMP*,*CoP*_ metric, additional filtering of CoP VFB significantly increased the physical shift initiation force applied by subjects, with the CoP_34_ VFB condition resulting in an average of 12% more and the CoP_58_ VFB condition resulting in an average of 23% more CoP NMP behavior compared to the CoP_10_ VFB condition. In terms of actual force, this corresponds to an average of 4.8N additional force for the CoP_34_ VFB condition and 12.9N additional force for the CoP_58_ VFB condition. These increases in shift initiation force are consistent with the reduced CoP shift time associated with increased VFB filtering. The effect of VFB condition on shift initiation force is likely caused by more than just the difference of attenuation in visually displayed NMP behavior.

These results show that differences in VFB delay and visually displayed NMP behavior result in significant differences in both shift speed and shift initiation force. Without separating these two elements, though, one cannot ascertain the individual effects of these two VFB characteristics on shift performance. Since VFB delay is known to have significant effects on static balance control [17,27], one might expect it to be the dominant VFB characteristic in the present examination of dynamic weight-shifting balance. Also, the duration of the NMP behavior is a portion of the total shift time (average of 0.64 s or 62 ± 12%) confined to the beginning of the shift, which could limit its effect on balance control compared to delay, which occurs over the entire duration of the shift. If delay is the dominant characteristic, the attenuation of displayed NMP behavior might have either complementary (increasing shift speed and NMP behavior) or opposite (decreasing shift speed and NMP behavior) effects, only less significant ones, with complementary effects being more likely. Future work might attempt to separate out the effects by constraining the CoP VFB to move exclusively toward the target after it appears, thereby eliminating all NMP behavior without generating additional time delay. Without some manipulation of the VFB, though, this approach would result in a step increase in the displayed CoP velocity from zero once the measured CoP passes through the initial shift position. Such an unexpected discontinuity in CoP velocity could lead to reduced reliance on the VFB, so one possible manipulation would be an anticipatory motion of the CoP VFB toward the target once that actual CoP began moving in that direction after the peak in NMP behavior.

### Non-minimum phase behavior provides additional information regarding weight-shifting balance performance

As in [15], the correlations between shift time and initial shift force reported in this study’s results were of intermediate magnitude, indicating that the shift speed and NMP ratio are not highly dependent metrics. While previous work has examined weight-shifting balance in terms of how quickly stroke patients are able to accomplish the task using metrics for shift speed [13] and the amount of weight that subjects can place upon a given side (i.e. shift distance) once the shift is accomplished [8], analysis of the force applied to initiate a dynamic weight shift has not been studied in clinical research. This lack of focus on shift initiation force is understandable, as the shift speed and shift distance metrics can be estimated in a clinical setting by therapists without the need for instrumentation, whereas estimation of shift initiation force requires use of a force plate. Recent implementation of low cost force plate balance tools in clinical systems [18], though, provides an opportunity for incorporating balance metrics such as NMP behavior, thereby expanding the means available for clinical balance assessment. For example, the NMP metric could be clinically relevant in situations in which the rehabilitation goal is for hemiparetic patients to increase the force applied through a weakened limb. Further studies examining this metric in the context of hemiparetic patients could provide support to the utility of the NMP metric for measuring asymmetry, as well as serving as a potential indicator of progress in rehabilitation.

### Subjects shifted more efficiently for later feedback conditions

While feedback conditions after the first ordinal position (first VFB provided to each subject) resulted in significantly increased *t*_*CoP*_ and decreased *r*_*NMP*,*CoP*_ values, no significant differences were seen for *t*_*VFB*_ and *r*_*NMP*,*VFB*_ due to ordinal position. This order effect may be explained by subjects becoming more adept at efficiently shifting based on the provided feedback, resulting in less effort spent as shown by slower and less forceful physical shifts without significantly affecting the feedback that was provided during those shifts with the various filters. This behavior was likely a result of the feedback progressing to each subsequent target location based on feedback shift time (*t*_*VFB*_), not physical shift time (*t*_*CoP*_) and is consistent with previous studies of dynamic balance tasks [16,28] that demonstrated improvement over time, indicating the presence of learning.

### Additional study required for clinical populations

Elderly subjects have been shown to complete weight shifting tasks more slowly and with greater difficulty than young adults [29]. Stroke patients, who are often advanced in years, are therefore likely to demonstrate differing results from younger subjects. As such, examination of an elderly subject pool would provide additional insight into VFB design in clinical applications. Additionally, a previous study showed no significant effects of VFB delay on static balance performance in stroke patients [30]. These differing results compared to those obtained from both young healthy [17] and elderly subjects [27] indicate that clinical subjects may be affected less by VFB delay. Such differences may be due to clinical subjects lacking the ability to utilize VFB to the same extent as healthy subjects and instead relying primarily on visual information from the environment as a result of cognitive deficits or the increased mental and physical effort necessary to simply remain standing. This idea is not necessarily inconsistent with previous work showing that stroke patients exhibit greater dependence on visual information in controlling their balance [31], which may be a result of impaired proprioception. In terms of a dynamic weight-shifting balance task incorporating changes in visually displayed NMP behavior, no previous work has examined the effects of filtering in dynamic weight shifting for clinical subjects. Indeed, due to this reduced effect of VFB delay on clinical subjects, such subjects could potentially see greater impact from the differences in NMP behavior provided in weight-shifting VFB and merit examination in a future study.

## Conclusions

This study demonstrated that increases in filtering applied to CoP feedback provided during a mediolateral weight-shifting balance task resulted in significantly increased shift initiation force and decreased physical shift times. As such, filtering of VFB in a weight-shifting task should be selected and reported accordingly in future work. These results show that simple filtering could be used to adjust VFB for balance rehabilitation tasks in a clinical setting in order to potentially optimize balance performance.

These significant effects of VFB filtering for young healthy subjects could potentially translate to differences for stroke patients. As such, modulation of VFB filtering could be used to provide subtle direction to patients performing a weight-shifting balance task. For example, lower functioning patients could be eased into performing weight-shifting balance activities with minimal VFB filtering to start, while higher functioning patients could be encouraged to shift with more vigor through increasing VFB filtering. For patients with hemiparesis, the magnitude of filtering could even be adjusted depending on the direction of the shift, thereby encouraging more forceful and quicker shifts to one direction compared to the other. The use of this filtering would likely be best applied on a subject-by-subject basis, with individual patient deficits and balance capabilities informing the therapist’s selection of the extent of VFB filtering.

## Supporting Information

S1 Text Supplementary data. (CSV)
